# Screening for Antileukemia Agents in FMS-like Tyrosine Kinase 3 (FLT3)-Mutated Acute Myeloid Leukemia Cells

**DOI:** 10.1021/acsptsci.5c00317

**Published:** 2025-07-22

**Authors:** Livia Bassani Lins de Miranda, Wítor Ribeiro Ferraz, Keli Lima, Jorge Antonio Elias Godoy Carlos, Fernando Moura Gatti, Rodrigo Heleno Alves, Gustavo Henrique Goulart Trossini, João Agostinho Machado-Neto

**Affiliations:** † Department of Pharmacology, Institute of Biomedical Sciences, University of Sao Paulo, São Paulo, CEP 05508-900, Brazil; ‡ Department of Pharmacy, School of Pharmaceutical Sciences, University of São Paulo, São Paulo, CEP 05508-000, Brazil; § Cancer Institute of the State of São Paulo, Faculdade de Medicina, University of São Paulo, São Paulo CEP 01246-000, Brazil

**Keywords:** antineoplastic agents, acute myeloid leukemia, FLT3 mutation, cell differentiation

## Abstract

Acute myeloid leukemia (AML) remains a challenging hematological malignancy due to its genetic heterogeneity, high relapse rates, and limited therapeutic options for refractory cases. FMS-like tyrosine kinase 3 (FLT3)-internal tandem duplication (FLT3-ITD) mutations are among the most frequent genetic alterations in AML, associated with poor prognosis and treatment resistance. In this study, we investigated the antileukemic potential of compound HI042, identified from a library of 78 molecules, focusing on its effects on FLT3-ITD-mutated AML models. HI042 selectively reduced the viability of FLT3-ITD-positive cell lines, induced apoptosis, disrupted cell cycle progression, and diminished the clonogenic potential. Chemoinformatics analysis revealed structural similarities between HI042 and retinoic acid analogues, known for their differentiation-inducing effects. Consistently, HI042 treatments increased the level of differentiation markers, including CD11b and transcription factors such as PU.1 and C/EBPs, particularly in MOLM-13 cells. Furthermore, combining HI042 with the FLT3 inhibitor quizartinib synergistically enhanced apoptosis and reduced cell proliferation. These findings highlight HI042’s dual activity in inducing differentiation and apoptosis while synergizing with established therapies. Overall, HI042 emerges as a promising candidate for targeted therapies against FLT3-ITD-mutated AML, addressing a critical need for novel treatment strategies for this high-risk AML subgroup.

## Graphical abstract

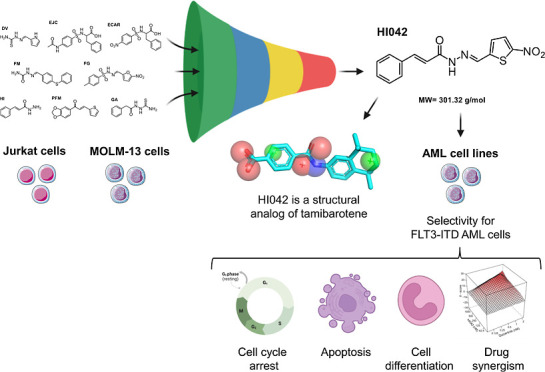

Acute myeloid leukemia (AML) is characterized by the uncontrolled proliferation of immature hematopoietic cells in the bone marrow or peripheral blood, resulting in impaired hematopoiesis.^1,2^ Studies have shown an increase in the incidence of AML, resulting in a global rise from 79,372 in 1990 to 144,645 in 2021, accompanied by an increase in the challenges involving the therapeutic handling of that burden.^2^ AML is the most incident subtype of leukemia and accounts for the highest percentage of death rates.^3^ The study of the genomic landscape of patients revealed AML as a heterogeneous disease, in which somatic mutations and cytogenetic factors impact prognosis and treatment outcomes.^4,5^

The application of genome sequencing led to the identification of a number of driver mutations, making it possible to understand the biology of AML pathogenesis.^6^ Alterations on the FMS-like tyrosine kinase 3 (FLT3) account for 30% of the AML mutations, in which the internal tandem duplication (FLT3-ITD) is the most common, often linked to poor prognosis.^6,7^ The FLT3-ITD mutation allows the activation of important signaling pathways independently of ligand binding, such as the phosphatidylinositol 3-kinase (PI3K)/AKT and MAPK/extracellular signal-regulated kinase (ERK) pathways.^8^ Over the past years, the research and development of FLT3 inhibitors and their study in clinical trials as single-agent or combination therapy.^9,10^ The use of FLT3 inhibitors has been demonstrated to be a promising treatment for relapsed and refractory AML patients. Currently, midostaurin, gilteritinib, and quizartinib are the only FDA-approved FLT3 inhibitors for use in the clinic.^10,11^ Despite the use of FLT3 inhibitors having improved patient outcomes, the emergence of resistance to those treatments represents a challenge.^7,12^

The increasingly high interest in targeted therapy evidence a shift in drug development for cancer treatment. The use of tyrosine kinase inhibitors is advantageous compared to traditional chemotherapy, as it targets cancer cells and not normal cells, increasing efficacy and decreasing toxicity and side effects.^13,14^ Imatinib was the first tyrosine kinase inhibitor developed, targeting the BCR::ABL1 oncoprotein and redefining the outcome for patients with chronic myeloid leukemia (CML). The approval of Imatinib by the FDA sparked research interest in small molecules and highlighted the importance of medicinal chemistry in drug discovery and development.^13,15,16^

The aim of the present study was to screen a single-center compound library to identify potential new antileukemic agents, particularly for FLT3-mutated AML models.

## Materials and Methods

### Cell Lines and Chemical Compound

The cell lines Jurkat, U-937, HEL, K-562, and KU812 were provided by Prof. Sara Teresinha Olalla Saad (Hemocentro, University of Campinas, Brazil). OCI-AML3, Kasumi-1, MOLM-13, and MV4-11 cells were provided by Prof. Eduardo Magalhães Rego (Faculdade de Medicina, University of São Paulo, Brazil). SET-2 cells were provided by Prof. Dr. Fabola Attié de Castro (School of Pharmaceutical Sciences of Ribeirão Preto, University of São Paulo, Ribeirão Preto, Brazil). The cell lines were cultivated in culture media recommended by the American Type Culture Collection (ATCC) or the Deutsche Sammlung von Mikroorganismen and Zellkulturen (DSMZ), supplemented with fetal bovine serum and penicillin/streptomycin. The cells were maintained at 37 °C in a 5% CO_2_ atmosphere. Compounds were synthesized in the Laboratory of Integration between Experimental and Computational Techniques (LITEC), diluted in dimethyl sulfoxide (Synth, Diadema, SP, Brazil), and stored at −20 °C (Table S1). The details of the synthesis of the compounds that were the focus of this study, HI042 and HI044, are described in the Supporting Information for publication.

### Cell Viability Assay

A total of 2 × 10^4^ cells per well were seeded in a 96-well plate in the appropriate medium in the presence of the vehicle or the molecules used for screening at concentrations of 1 and 10 μM for 72 hours in the initial screening. Later, the cells were treated with vehicle or different concentrations of HI042 or HI044 (0.3, 0.6, 1.25, 2.5, 5, 10, 20, and 40 μM) for 24, 48, and 72 h. For the synergism analysis, MV4-11 and MOLM-13 cells were treated with HI042 (0.06–1 μM) and quizartinib (0.12–2 nM), either individually or in combination. The ZIP synergy score was calculated using the SynergyFinder software (https://synergyfinder.fimm.fi/). Next, 10 μL of methylthiazole tetrazolium (MTT, Sigma-Aldrich) solution (5 mg/mL) was added and incubated at 37 °C with 5% CO_2_ for 4 hours. The reaction was stopped using 100 μL of 0.1 N HCl in anhydrous isopropanol. The absorbance at 570 nm was used to evaluate cell viability. The IC_50_ values were calculated by nonlinear regression analysis using GraphPad Prism 8 (GraphPad Software, Inc., San Diego, CA, USA).

### Autonomous Colony Formation Assay

A total of 1 × 10^3^/mL cells were seeded in a semisolid methylcellulose medium (MethoCult 4230; StemCell Technologies Inc., Vancouver, BC, Canada) and treated with the vehicle or HI042 (0.3, 0.6, 1.25, 2.5, and 5 μM). After 7–10 days of culture, colonies were detected by adding 100 μL of MTT reagent (5 mg/mL) and quantified using ImageJ software (U.S. National Institutes of Health, Bethesda, MD, USA).

### Cell Cycle Analysis

A total of 1 × 10^5^ cells per well were seeded in a 24-well plate and treated with vehicle or HI042 (0.5, 1, 2, and 4 μM). After 72 h, the cells were fixed with 70% ethanol and stored at 4 °C for at least 2 h. Next, the cells were stained with 20 μg/mL propidium iodide (PI) containing 10 μg/mL RNase A and incubated for 30 min in a light-protected area. The DNA content distribution was determined by using flow cytometry (FACSCalibur; Becton Dickinson, Franklin Lakes, NJ, USA) and analyzed using FlowJo software (Treestar, Inc., San Carlos, CA, USA).

### Apoptosis Assay

A total of 1 × 10^5^ cells per well were seeded in a 24-well plate and treated with the vehicle or HI042 (0.25, 0.5, 1, 2, and 4 μM) for 72 h. The cells were then washed with ice-cold phosphate-buffered saline (PBS) and resuspended in a binding buffer containing 1 μg/mL propidium iodide (PI) and 1 μg/mL APC-labeled annexin V (BD Pharmingen, San Diego, CA, USA). The cells were incubated in a light-protected area for 15 min at room temperature. For each sample, 10,000 events were acquired on a FACSCalibur (Becton Dickinson, Lincoln *P*ark, NJ, USA) and analyzed with FlowJo software vX.0.7 (Treestar, Inc., San Carlos, CA, USA).

### Western Blot Assay

A total of 2 × 10^6^ cells were seeded and treated with the vehicle or HI042 (0.25, 0.5, and 1 μM). After 72 h, the cells were collected and lysed with extraction buffer (10 mM EDTA, 100 mM Tris, 10 nM Na_4_P_2_O_7_, 100 mM NaF, 10 mM Na_3_VO_4_, 2 mM phenylmethane sulfonyl fluoride, 1% Triton X-100). Equal amounts of total protein extract were used, followed by SDS-PAGE and Western blot analysis with antibodies against PARP1 (#9542), γH2AX (#9718), and α-tubulin (#2144) (Cell Signaling Technology), and the SuperSignal West Dura Extended Duration Substrate system (Thermo Fisher Scientific, Waltham, MA, USA) and the G:BOX Chemi XX6 gel documentation system (Syngene, Cambridge, United Kingdom) were used.

### Cheminformatics Analysis

The molecule HI042 was drawn using ChemDraw Professional V.2023 (PerkinElmer, MA, USA) and converted to a 3D structure. It was energy-minimized by employing the Merck Molecular Force Field (MMFF) with implicit model molecules of water using Spartan software (Wave function, Inc., CA, USA). The 3D structure model was applied to conduct a similarity search for HI042 in the virtual database SciFinder (Chemical Abstracts Service, OH, USA) with cutoff filters set to 80–84% similarity, focusing on commercially available molecules. Simultaneously, a search in the online database ChEMBL (European Molecular Biology Laboratory, Heidelberg, Germany) for “small molecules” tagged with “leukemia” was made. The data set (DS) files were saved in.sdf and .xls formats and processed with OpenEye’s Omega (OpenEye Scientific, NM, USA) software. SMILES (Simplified Molecular-Input Line-Entry System) from both data sets were normalized to the “aromatic” format via the National Center for Biotechnology Information (NCBI) CACTUS (Chemical Access and Control Tool for Unified Systems) platform and converted to .sdf format using RDKit (RDKit community, worldwide). The final analysis data set (FDS), comprising the merged SciFinder and ChEMBL, and a subset of analyses exclusively with ChEMBL-derived molecules (CDS), was used in the similarity search analyses. All molecules in FDS and CDS underwent MMFF-based energy minimization, as performed for HI042. A shape-based strategy was employed for the structural similarity analysis. For it, two types of ligand-based drug design studies were conducted: a pharmacophore-based molecular similarity screening using OpenEye’s vROCS (OpenEye Scientific, NM, USA) software and an electronic similarity screening using OpenEye’s EON (OpenEye Scientific, NM, USA) software. Both screenings were performed with maximum accuracy. Each similarity search was conducted in triplicate for FDS and CDS, resulting in 12 individual screenings. Results from vROCS were ranked using the “Tanimoto Combo”, incorporating molecular shape similarity, and “Tanimoto Color”, which measures pharmacophore feature overlap alongside structural overlap assessments. EON results were ranked based on “EON Combo”, which evaluates both shape and electrostatic similarity, supplemented by isolated Tanimoto Shape indices. Visual inspections of molecular and pharmacophore alignments further supported the evaluations. The top 20 ranked molecules from each triplicate were selected as cutoff criteria. Finally, the best five molecules from each screening type (FDS-vROCS, FDS-EON, CDS-vROCS, and CDS-EON) were included in the final results, totaling 20 molecules, along with those identified as consensus across different similarity search methods.

### Cell Differentiation Analysis

A total of 1 × 10^5^ cells per well were seeded in a 24-well plate and treated with the vehicle or HI042 (0.25, 0.5, and 1 μM) for 72 h. The differentiation rate was determined by evaluating the population of CD11b-positive cells (percentage and MFI levels). Experiments were conducted using flow cytometry (FACSCalibur; Becton Dickinson) and analyzed using FlowJo software (Treestar, Inc.).

### Gene Expression Analysis

Total RNA from MOLM-13 and MV4-11 cells treated with vehicle or HI042 (1 μM) for 72 h was extracted using TRIzol reagent (Thermo Fisher Scientific) and reverse-transcribed using the High-Capacity cDNA Reverse Transcription Kit (Thermo Fisher Scientific). Quantitative PCR (qPCR) was performed using a QuantStudio 3 Real-Time PCR System in conjunction with the SYBR Green system (Thermo Fisher Scientific) and specific primers for *SPI1* (FW: ATGAAGGACAGCATCTGGTGG, RV: TTCACCTTCTTGACCTCGCCC), *CEBPA* (FW: GCAAACTCACCGCTCCAATG, RV: TTCTCTCATGGGGGTCTGCT), and *CEBPB* (FW: AGAAGACCGTGGACAAGCACAG, RV: CTCCAGGACCTTGTGCTGCGT). *HPRT1* (FW: GAACGTCTTGCTCGAGATGTGA, RV: TCCAGCAGGTCAGCAAAGAAT) and *ACTB* (FW: AGGCCAACCGCGAGAAG, RV: ACAGCCTGGATAGCAACGTACA) were used as reference genes. The relative quantification value was calculated using the 2^–ΔΔCT^ method.^17^ A negative “No Template Control” was included for each primer pair.

### Statistical Analysis

Statistical analyses were performed using GraphPad Prism 8 (GraphPad Software). For comparisons, Student’s *t* tests or ANOVA with Bonferroni post-tests were used. At least three independent experiments were conducted for each method. A *p*-value of <0.05 was considered statistically significant.

## Results

### Screening for Compounds with Antileukemia Activity in FLT3-Mutated AML Models

First, two leukemia cell lines, Jurkat (ALL) and MOLM-13 (AML), were utilized for the initial screening of the compounds. Three out of 78 compounds reduced cell viability at a concentration of 1 μM, specifically FM017 for Jurkat cells and HI042 and HI044 for MOLM-13 cells (Figure 1A). Due to the selectivity of HI042 and HI044 for MOLM-13 cells, both were used for further cell viability screening with additional AML experimental models. Most of the cell lines, except K-562, showed sensitivity to treatment with HI044, with IC_50_ values ranging from 0.19 to 2.37 μM. On the other hand, only three out of nine leukemia cell lines were sensitive to HI042, with IC_50_ values of 0.62 μM for MOLM-13, 0.33 μM for MV4-11, and 0.89 μM for OCI-AML3 cells (Figure 1B). Due to its apparent selectivity, compound HI042 was chosen for further analysis in FLT3-mutated AML cell lines.

**Figure 1:**
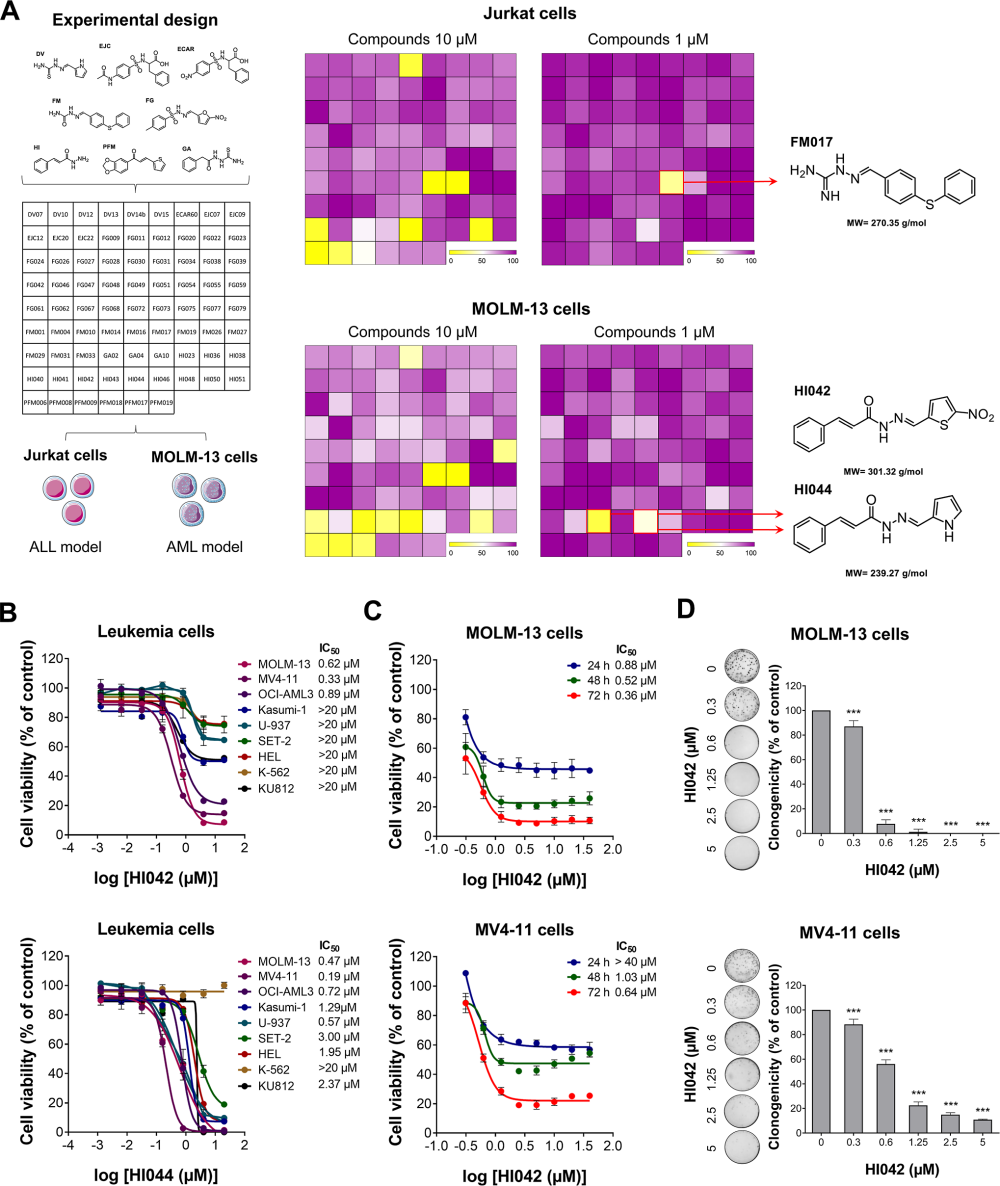
Screening for compounds with antileukemia activity in FLT3-mutated acute myeloid leukemia models. (A) Experimental design of the drug screening with 78 compounds. Jurkat and MOLM-13 cells were treated with all compounds at concentrations of 1 and 10 μM. The heatmap indicates cell viability upon treatment with the compounds, highlighting FM017, HI042, and HI044 as the most efficient compounds. (B) Concentration-dependent cytotoxicity was assessed by the MTT assay. MOLM-13, MV4-11, OCI-AML3, Kasumi-1, U-937, SET-2, HEL, K-562, and KU812 were treated with the vehicle, HI042, or HI044 at concentrations of 0.001, 0.006, 0.032, 0.16, 0.8, 1.6, 4, and 20 μM for 72 h. (C) Concentration- and time-dependent viability assays of MOLM-13 and MV4-11 cells upon treatment with the vehicle or HI042 at concentrations of 0.312, 0.625, 1.25, 2.5, 5, 10, 20, and 40 μM for 24, 48, and 72 h. (D) For the autonomous colony formation assay, MOLM-13 and MV4-11 cells were treated with the vehicle or HI042 (0.3, 0.6, 1.25, 2.5, and 5 μM) for 7–10 days. The bar graph represents the mean ± SD of the relative number of colonies (% of control). ***p < 0.001; ANOVA and Bonferroni post-test.

To determine viability in a time- and concentration-dependent manner, FLT3-mutated AML cells were treated with HI042 for different durations of drug exposure. The IC_50_ values for MOLM-13 cells were 0.88, 0.52, and 0.36 μM and >40, 1.03, and 0.64 μM for MV4-11 cells for the periods of 24, 48, and 72 h, respectively (Figure 1C), indicating that the effects of HI042 on the reduction of cell viability are time-dependent. Notably, HI042 also reduced autonomous clonal growth in FLT3-mutated AML cells in a concentration-dependent manner (Figure 1D). Based on these results and the doubling time of the cell models, a time of 72 h was selected for the subsequent experiments.

### HI042 Induces Cell Death and Disrupts Cell Cycle Progression on FLT3-Mutated AML Cells

To further assess the effects of treatment with the molecule on the cells that showed sensitivity to HI042, we evaluated the cell cycle progression and apoptosis. The cell cycle progression analysis revealed an increase in the _Sub_G1 cell population in MOLM-13 and MV4-11 cells, indicating cell death (Figure 2A). Both MOLM-13 and MV4–11 cells exhibited an increase in apoptosis, as indicated by the rise in annexin V^+^ cell populations (Figure 2B,C), which was confirmed by increased PARP1 cleavage and γH2AX expression (Figure 2D).

**Figure 2:**
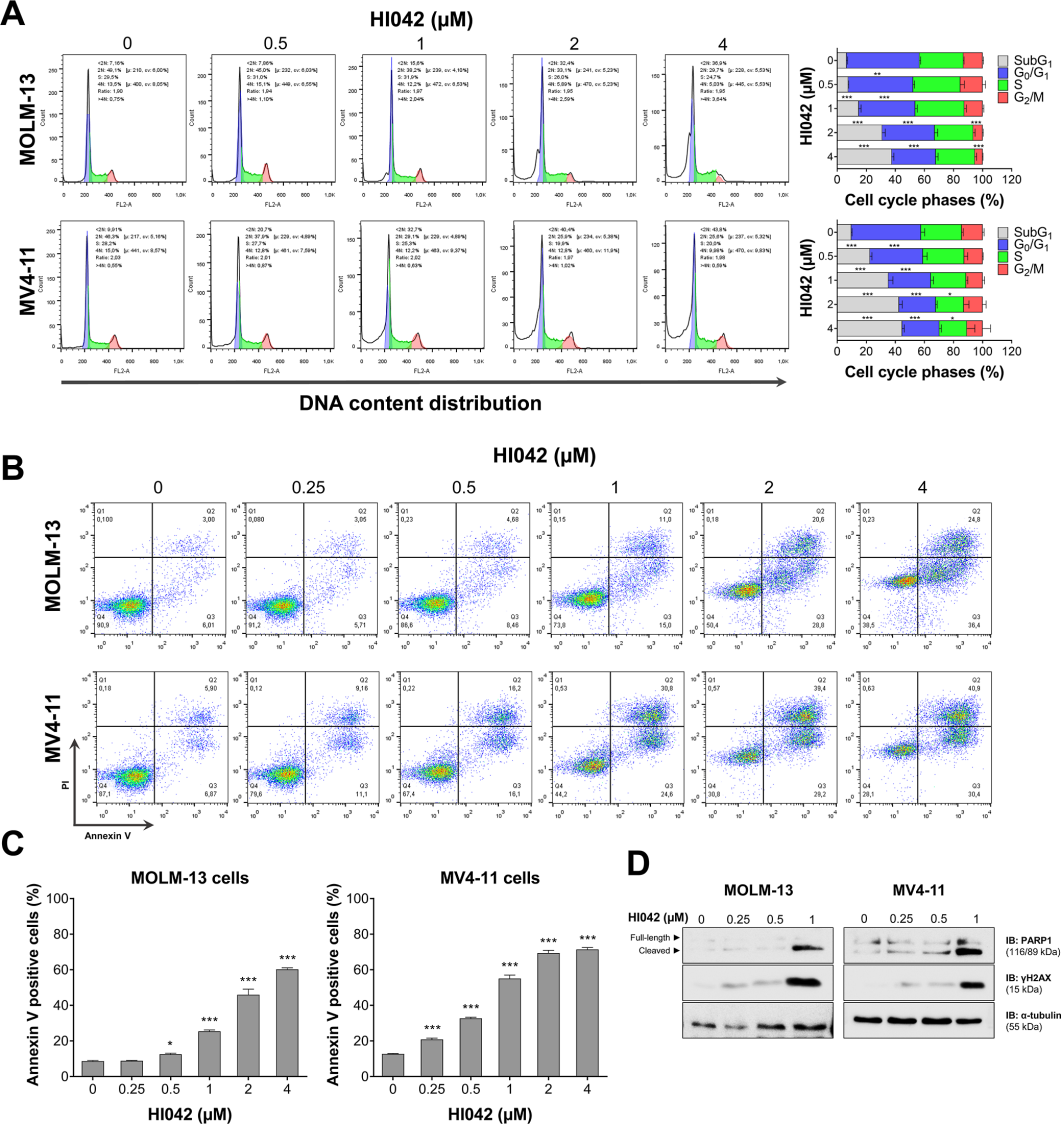
HI042 induces cell death in FLT3-mutated AML cells. (A) Cell cycle progression was determined by PI staining using flow cytometry. Cells were treated with the vehicle or HI042 (0.5, 1, 2, and 4 μM) for 72 h. A representative histogram for each condition is illustrated. Bar graphs represent the mean ± SD of the percentage of cells in subG_1_, G_0_/G_1_, S, G_2_/M, and >4 N cells. *p < 0.05, **p < 0.01, and ***p < 0.001; ANOVA and Bonferroni post-test. (B) Apoptosis was assessed by annexin V/PI staining using flow cytometry. MOLM-13 and MV4-11 cells were treated with the vehicle or HI042 (0.25, 0.5, 1, 2, and 4 μM) for 72 h. The representative dot plots are shown for each condition; the cell death population (annexin V-positive cells) is in the upper and lower right quadrants (Q2 plus Q3). (C) Bar graphs represent the mean ± SD of at least three independent experiments, *p < 0.05, ***p < 0.001; ANOVA and Bonferroni post-test. (D) Western blot analysis for PARP1 and γH2AX in total cell extracts from MOLM-13 and MV4-11 cells treated with the vehicle or HI042 (0.25, 0.5, and 1 μM) for 72 h.

### HI042 Is a Structural Analogue of the Antileukemia Drug Tamibarotene

An initial compound search was conducted using the SciFinder database, resulting in the identification of 152 small molecules. The data set was exported in both Structure Data File (.sdf) and Excel spreadsheet (.xls) formats for subsequent analysis. Subsequently, 55 molecules were excluded due to distorted SMILES, erroneous 3D structures, or incorrect similarity indices. The resulting curated set, herein referred to as the SciFinder data set, comprised 97 chemically valid molecules.

Simultaneously, a structure- and target-oriented search was carried out in the ChEMBL database, focusing on compounds annotated with activity against leukemia-specific biochemical targets. This effort identified 512 molecules, which were exported in .sdf and .xls formats and subsequently processed using the Omega software package (OpenEye Scientific Software, NM, USA) to generate 3D conformations.

An additional curation step was conducted, leading to the exclusion of 20 compounds due to predicted toxicity alerts, coordination compounds, or inconsistencies in similarity indexing. The resulting data set comprised 492 compounds, herein referred to as the ChEMBL-derived data set (CDS). The SciFinder and CDS data sets were merged, resulting in a unified final data set (FDS) containing 590 compounds.

Ligand-based pharmacophore modeling is widely employed to identify common structural and chemical features of active compounds, which can be used to screen for novel bioactive molecules or to perform similarity searches aimed at identifying known drugs with similar mechanisms of action.^18^ In our study, pharmacophore modeling was conducted to identify the key chemical features of HI042 that may contribute to its biological activity. The analysis was performed by using the vROCS platform (OpenEye Scientific Software), yielding a consensus pharmacophore hypothesis comprising seven pharmacophoric features: four hydrogen bond acceptors, one hydrogen bond donor, and two aromatic ring features (Figure S1).

The similarity search analyses show high reproducibility and consistency across all triplicates for both vROCS and EON methods applied to the FDS and CDS data sets. For vROCS, the ranking was based on the Tanimoto Combo index, which considers both 3D shape similarity and pharmacophore overlap. EON ranking was determined using the EON Combo index, which evaluated Tanimoto similarity for molecular shape and electrostatic properties. Additionally, structural overlap metrics were considered in the final evaluations. The vROCS analyses of FDS included only molecules from SciFinder with 80 – 84% similarity to HI042, while EON analyses were less restrictive, ranking molecules from ChEMBL with bioactivity against leukemia. Notably, the top five ranked molecules in the EON analysis of CDS also appeared among the top 20 for DBF.

All triplicates of the vROCS and EON analyses exhibited identical results, further confirming the robustness of the methodology. The five top-ranked molecules in the vROCS analysis of DBF were identified with strong molecular alignments and shape similarity. Similarly, the EON analysis highlighted compounds with a significant electrostatic similarity to HI042. Among these, three molecules—506, 521, and 574—consistently appeared among the top 20 in both vROCS and EON screenings, confirming their high theoretical structural and electronic similarity to HI042. Their ranking positions and similarity metrics were carefully analyzed to determine their suitability as lead compounds.

Three molecules were identified as consensus hits, consistently ranking among the top 20 in vROCS and EON analyses. These molecules, derived from the SciFinder data set, exhibited high structural and electronic similarity to HI042. Based on the results of our ranking models, supported by visual inspection, four candidate molecules were prioritized for subsequent stages of the study. Tamibarotene (prevenient from the EON and vROCS analyses of DBC, and the consensus hits), a retinoic acid analogue, demonstrated the highest ranking and alignment across structural and pharmacophore overlaps within the CDS data set and is reported to have activity against acute promyelocytic leukemia (APL), and its biological target is the retinoic acid receptor alpha (RARα).^19–21^ Two consensus molecules showed strong structural and electronic similarity to HI042, with one exhibiting defined antibiotic and antitrypanosomal activity and the other displaying antibiotic and antifungal properties. The third molecule, the top-ranked compound from the vROCS analysis of FDS, demonstrated excellent alignment and overlap properties but lacked any reported biological activity.

These findings reinforce the effectiveness of the applied similarity analysis strategies, emphasizing the importance of integrating molecular shape and electrostatic similarity analyses in ligand-based drug discovery approaches. The high reproducibility of the screening results, coupled with the observed structural consistencies, highlights the potential of the selected compounds for further experimental validation. Among the four selected compounds, only one, tamibarotene, demonstrated antileukemic activity.^22^ Figure S1 illustrates the molecular alignment between HI042 and tamibarotene, highlighting the superimposed pharmacophore features.

### HI042 Induces Cell Differentiation in FLT3-Mutated AML Cells

Due to the structural similarity between HI042 and a retinoic acid analogue, analyses to verify similarities in effects were carried out with the FLT3-mutated cell lines. First, CD11b-positive cell populations were analyzed by flow cytometry. Results indicate that both MOLM-13 and MV4-11 show an increase in the fold change of CD11b-positive cells of approximately 3-fold and 2-fold, respectively (Figure 3A). Gene expression analysis of HI042-treated cells revealed a significant increase in the expression of the genes *CEBPA* and *CEBPB* in MOLM-13 cells but not in MV4-11 (Figure 3B).

**Figure 3:**
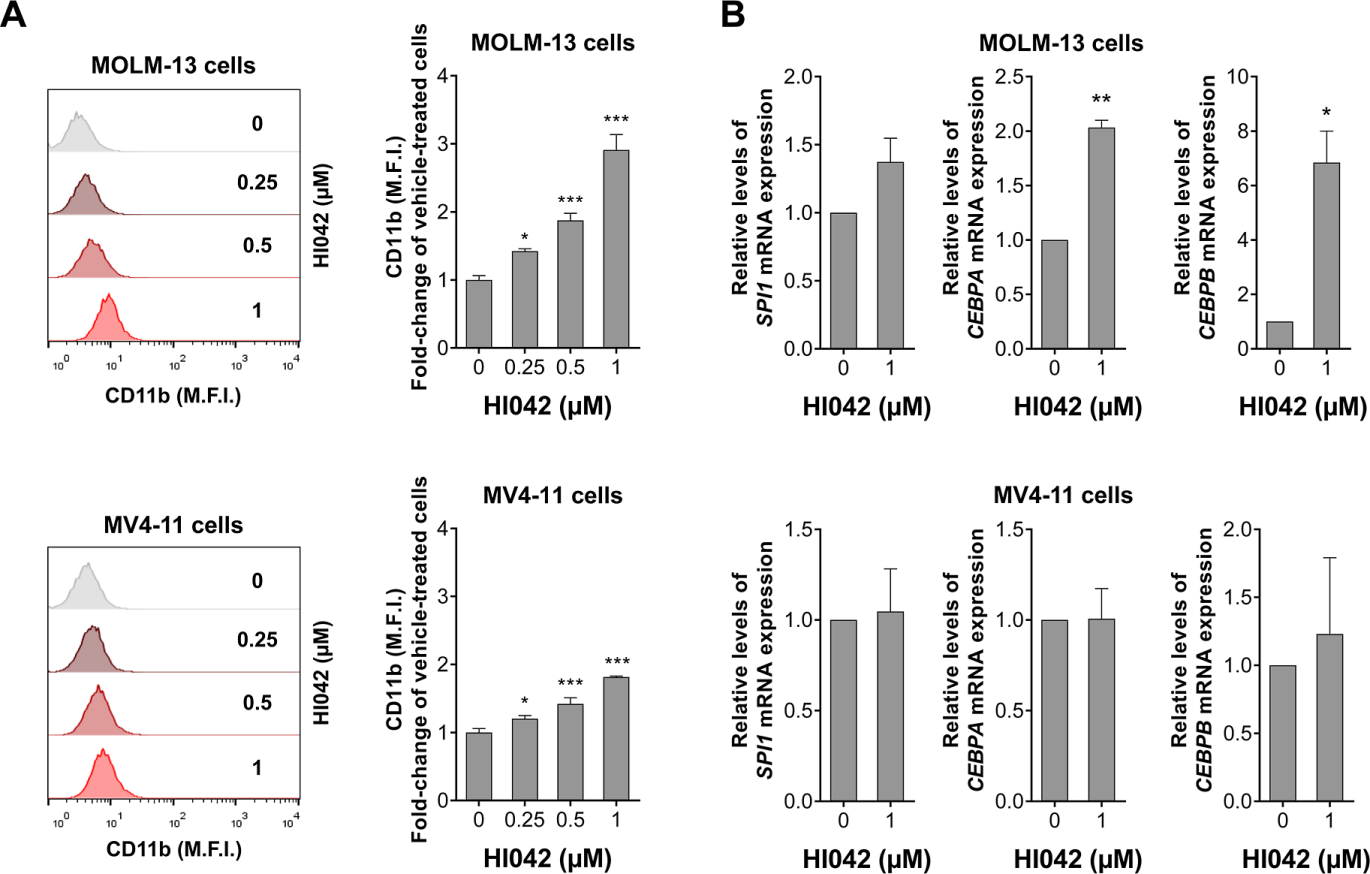
HI042 induces cell differentiation in MOLM-13 and MV4-11 cells. (A) MOLM-13 and MV4-11 cells were exposed to the vehicle or HI042 (0.25, 0.5, and 1 μM) for 72 h. The histograms represent the mean fluorescence intensity (M.F.I.) for PE-CD11b (left). Bar graphs represent the mean ± SD of at least three independent experiments. The p-values are indicated; *p < 0.05, ***p < 0.001; ANOVA and Bonferroni post-test. (B) The bar graph represents the mean ± SD of the fold change of vehicle-treated cells for significantly modulated genes in FLT3-mutated AML cells upon exposure to HI042. *p < 0.05; **p < 0.01; Student’s *t*-test.

### HI042 Synergizes with Quizartinib to Reduce the Viability of MV4-11 and MOLM-13 Cells

Given that HI042 appears to exhibit antileukemic activity similar to a retinoic acid analogue and that previous studies have demonstrated that the combination of all-trans retinoic acid (ATRA) and FLT3 inhibitors enhances antineoplastic effects,^23^ we investigated the effects of HI042 on quizartinib-induced cell viability reduction in FLT3-mutated AML cells. Our findings indicate that HI042 acts synergistically with quizartinib to reduce the viability of MV4-11 (ZIP synergy score = 8.11) and MOLM-13 (ZIP synergy score = 5.23) cells. For instance, in MV4-11 cells, HI042 (0.5 μM) and quizartinib (1 nM) individually reduce viability by 17.2 ± 1.1% and 35.9 ± 2.6%, respectively, but their combination reduces viability by 62.8 ± 3.7% (all *p* < 0.05). Similarly, in MOLM-13 cells, HI042 (1 μM) and quizartinib (2 nM) individually reduce viability by 52.3 ± 4% and 34.5 ± 1.9%, respectively, but their combination reduces viability by 88.7 ± 2.8% (all *p* < 0.05, Figure 4).

**Figure 4:**
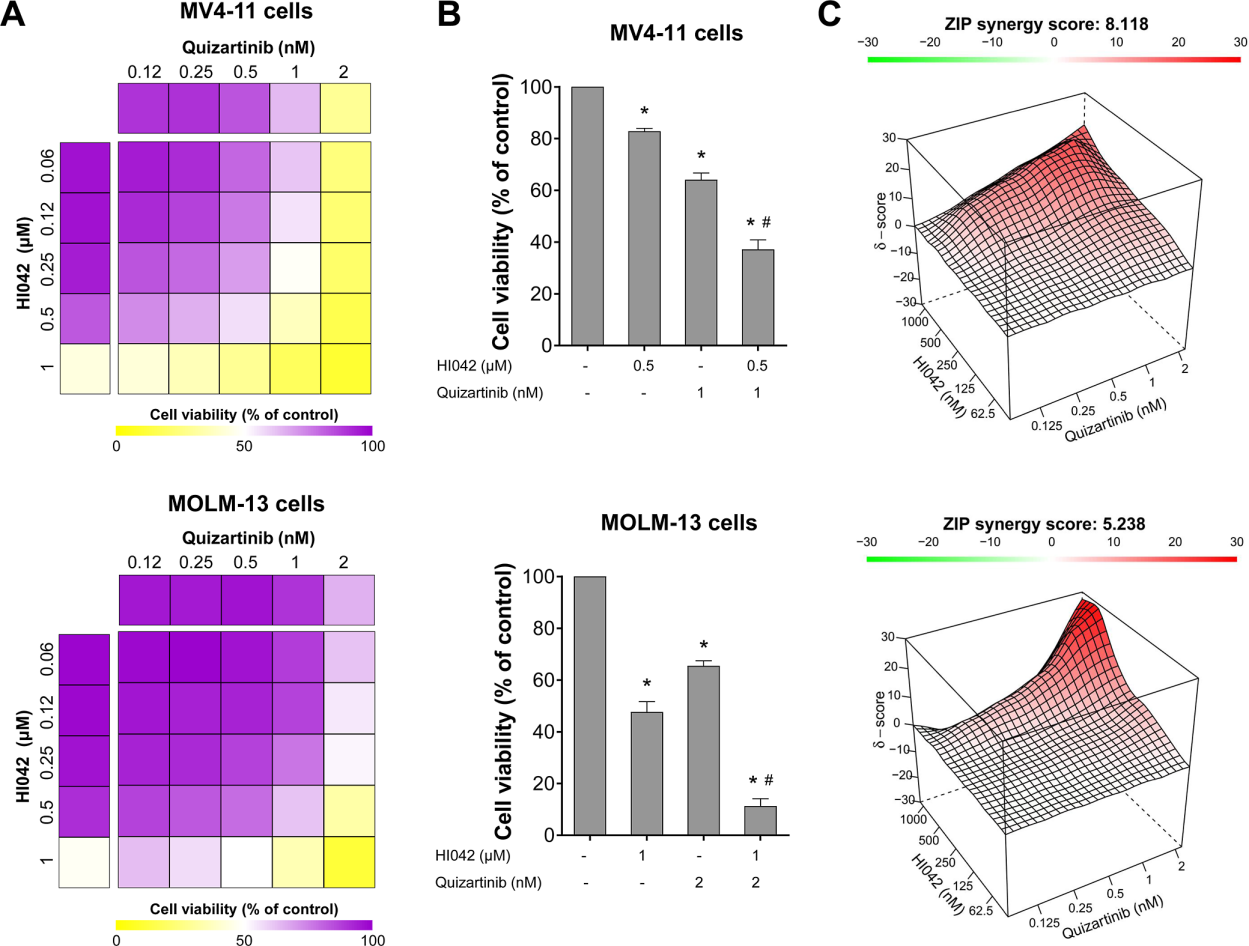
HI042 and quizartinib act synergistically to reduce the viability of MV4-11 and MOLM-13 cells. (A) Dose–response cytotoxicity for HI042 and quizartinib was analyzed using the methylthiazolyl tetrazolium (MTT) assay in MV4-11 and MOLM-13 cells. Cells were exposed to the vehicle or graded concentrations of HI042 and quizartinib, either alone or in combination, for 72 h, as indicated. Values are expressed as percentages relative to vehicle-treated cells. (B) Bar graphs illustrate context-relevant combinations. *p < 0.05 for treatment versus the vehicle, and #*p* < 0.05 for monotherapy versus combination therapy; ANOVA test with the Bonferroni post-test. (C) The ZIP synergy score was calculated using the SynergyFinder software (https://synergyfinder.fimm.fi/). Results are presented as the mean of at least four independent experiments.

## Discussion

The arsenal of treatment options for AML patients has greatly improved over the last 10 years, and a better understanding of molecular and pathophysiological characteristics of the disease has led to the development of several new agents. Despite the advances that the approval of new therapies provided, AML treatments remain a challenge for reasons such as genetic heterogeneity, which makes patients’ responses often hard to predict, and the incidence of relapsed/refractory AML.^24,25^ Long-term disease-free survival after standard chemotherapy is around 40–50%, meaning that around 60–40% of the patients fail to achieve remission and develop refractory AML, drastically limiting the options of treatments for patients.^26,27^ As for the FLT3 inhibitors, first-generation molecules midostaurin and sorafenib have been approved in first-line therapy combined with intensive chemotherapy for FLT3-mutated patients; however, they present little activity in relapsed AML. The next-generation inhibitors quizartinib and gilteritinib, on the other hand, have been described as effective in treating relapsed or refractory patients by multiple clinical trials, particularly in individuals older than 18 years.^28–31^ Notwithstanding advancements and promising results achieved with treatments with FLT3 inhibitors, the rapid emergence of resistance presents a challenge by shortening the duration of clinical response.^31,32^

In this study, a library containing 78 compounds was used to treat MOLM-13 and Jurkat cells, models for acute myeloid and lymphoblastic leukemias, respectively. HI042 and HI044 were potent in reducing the cell viability of the AML model. Next, viability assays using a molecularly heterogeneous panel of AML cell lines demonstrated a certain selectivity of the HI042 molecule toward the models with the FLT3-ITD mutation. The compound was chosen as the object of study for that reason. Further assays utilizing the two most sensitive cell lines, MOLM-13 and MV4-11, demonstrated the antineoplastic potential of treatments with HI042 by the decrease of clonogenic ability and inducing cell death, evidenced by the SubG1 population on cell cycle progression and an increase of annexin V-positive cell populations. Protein expression analysis confirmed the findings by the cleavage of PARP1 and increased expression of γH2AX, indicators of apoptosis and DNA damage, respectively.^33,34^

Chemoinformatics analysis demonstrated structural similarities between HI042 and a retinoic acid analogue. ATRA is a compound synthesized from retinol and has been used as a therapy option for APL.^35,36^ The binding of ATRA to the RARα promotes differentiation in myeloid cells.^36^ ATRA-based therapies became a topic of research on non-APL AML models as a way to induce differentiation and lead the cells to apoptosis. Studies show that the retinoic acid analogue exhibits antineoplastic effects on FLT3-ITD-mutated AML cells by inducing apoptosis and cell cycle arrest and apoptosis and additionally downregulates CHK1 proteins, which results in mitotic catastrophe.^37^ Furthermore, treatment combinations of ATRA and arsenic trioxide reportedly inhibit FLT3 signaling pathways, displaying synergic cytotoxic effects.^38,39^ To investigate whether HI042 also induces differentiation, cell populations CD11b^+^ were analyzed with a flow cytometer. CD11b is a cell marker located on the cell surface that indicates differentiation of the myeloid-monocytic lineage.^40^ Treatments with HI042 on MOLM-13 cells showed a higher increase in the fold change of the CD11b marker compared to MV4–11 cells. Gene expression analysis revealed that upon treatment with HI042, MOLM-13 cells presented an increase in the expression of the markers *SPI1*, which encodes PU.1, a transcription factor with an essential role in hematopoietic stem cell differentiation.^41^ Additionally, treatments increased the expression of the genes *CEBPA* and *CEBPB*, which encode the transcription factors C/EBPα and C/EBPβ, regulators of myeloid lineage commitment and stimulators for granulocyte differentiation.^42,43^ Interestingly, the genes were not significantly modulated in MV4-11 cells.

Moreover, previous works have demonstrated the synergistic effects when ATRA is utilized combined with FLT3 inhibitors in cell models with the FLT3-ITD mutation.^23,44^ Combinations with ATRA and sorafenib increased cell death and decreased clonogenic ability in cell lines and patient samples, as well as improved disease progression and survival in mouse models.^44^ Additionally, pharmacological combinations of ATRA and quizartinib result in a synergistic increase of apoptosis and inhibition of the FLT3 signaling pathway in cell models.^23^ Consistent with the literature, treatments with HI042 combined with quizartinib resulted in synergistic effects enhancing cell death of MOLM-13 and MV4-11 cells.

Tamibarotene, a synthetic retinoid, shares structural, electronic features with HI042, as suggested in the similarity search analyses.^45^ Both exhibit a planar aromatic framework that facilitates π–π interactions along with hydrogen bond donor and acceptor groups that are critical for interacting with the target. Molecular similarity assessment is a widely used strategy in drug discovery to identify new compounds with potential biological activity similar to known molecules or suggest the same mechanism of action to compounds that present common structural features. Previous studies have shown that high structural and electronic similarity indices are strong indicators of potential interactions with similar molecular targets. In this context, alignment and overlap analyses between HI042 and tamibarotene, conducted using vROCS and EON tools, suggest a high degree of concordance in terms of the three-dimensional molecular shape and electrostatic properties. This supports the hypothesis that HI042 may interact with targets similar to tamibarotene, including the RARα, crucial in regulating gene transcription and cell differentiation.^46,47^

The target fishing approach, essential for drug repurposing studies and the discovery of new therapeutic applications, was employed to predict the potential molecular targets of HI042. Molecular and pharmacophore similarity-based strategies were employed to identify previously known targets for tamibarotene and generate hypotheses regarding potential interactions with HI042. The methodological robustness of this study was ensured through the multiple molecular similarity approaches in triplicate, highlighting the consistency of the obtained results.^48^ Therefore, the data gathered reinforce the hypothesis that HI042 may act on biological pathways similar to those of tamibarotene and open possibilities for exploring these therapeutic targets to improve the activity of new compounds. Experimental validation of these predictions could establish HI042 as a novel bioactive entity, contributing to advancements in treating diseases such as APL and other pathologies associated with retinoic acid signaling.^48,49^

In conclusion, this study highlights the antileukemic potential of the HI042 molecule, particularly in FLT3-mutated AML models, identified from a library of 78 compounds. HI042 exhibited selective activity against FLT3-ITD cell lines, inducing apoptosis, suppressing proliferation, and disrupting the cell cycle. Chemoinformatics analysis revealed structural similarities between HI042 and a retinoic acid analogue, which is known to promote differentiation in myeloid cells. Consistently, in vitro assays demonstrated HI042’s ability to induce differentiation, evidenced by increased CD11b expression and upregulation of genes associated with myeloid commitment, such as SPI1, CEBPA, and CEBPB. Furthermore, HI042 showed synergistic effects with quizartinib, enhancing apoptosis and reducing clonogenicity in FLT3-ITD models.

## Supplementary Material



## Notes

Chemistry characterization, three-dimensional representation of the HI042 molecule and its pharmacophore, along with its alignment with tamibarotene, and list of compounds tested and the effects observed (PDF)

## Abbreviations

ALL: acute lymphoblastic leukemia
AML: acute myeloid leukemia
APL: acute promyelocytic leukemia
ATCC: American Type Culture Collection
ATRA: all-trans retinoic acid
CML: chronic myeloid leukemia
DSMZ: Deutsche Sammlung von Mikroorganismen and Zellkulturen
ERK: extracellular signal-regulated kinase
FDA: Food and Drug Administration
FLT3: FMS-like tyrosine kinase 3
FLT3-ITD: FMS-like tyrosine kinase 3-internal tandem duplication
LBDD: ligand-based drug design
MMFF: Merck Molecular Force Field
PI: propidium iodide
PI3K: phosphatidylinositol 3-kinase
qPCR: quantitative PCR
RARα: retinoic acid receptor alpha.
