# Supplementary material for: Screening for Antileukemia Agents in FMS-like Tyrosine Kinase 3 (FLT3)-Mutated Acute Myeloid Leukemia Cells
Source: ACS Pharmacol Transl Sci. 2025 Jul 22;8(8):2756–66. doi: 10.1021/acsptsci.5c00317 (PMC12340635; doi:10.1021/acsptsci.5c00317)
Supplement: Supplementary file 1 [file pt5c00317_si_001.pdf]

Supporting Information

Screening for antileukemia agents in FMS-like tyrosine kinase 3 (FLT3)-mutated acute myeloid leukemia cells

Livia Bassani Lins de Miranda[1], Wítor Ribeiro Ferraz[2], Keli Lima[1,3], Jorge Antonio Elias Godoy Carlos[1], Fernando Moura Gatti[2], Rodrigo Heleno Alves[2], Gustavo Henrique Goulart Trossini[2,*], João Agostinho Machado-Neto[1,*]

[1] Department of Pharmacology, Institute of Biomedical Sciences, University of Sao Paulo, São Paulo, CEP 05508-900, Brazil
[2] Department of Pharmacy, School of Pharmaceutical Sciences, University of São Paulo, São Paulo, CEP 05508-000, Brazil
[3] Cancer Institute of the State of São Paulo, Faculdade de Medicina, University of São Paulo, São Paulo, CEP 01246-000, Brazil

***Corresponding authors:**
Gustavo Henrique Goulart Trossini, PhD
Department of Pharmacy
School of Pharmaceutical Sciences of University of São Paulo
Av. Prof. Lineu Prestes, 580, CEP 05508-000, São Paulo, SP, Brazil
Phone: 55-11-3091-3674
Email: trossini@usp.br

João Agostinho Machado-Neto, PhD
Department of Pharmacology
Institute of Biomedical Sciences of University of São Paulo
Av. Prof. Lineu Prestes, 1524, CEP 05508-900, São Paulo, SP, Brazil
Phone: 55-11-3091-7467; Fax: 55-11-3091-7322
Email: jamachadoneto@usp.br

Table of contents

Supplementary material and methods	S-2
Figure S1	S-3
Table S1	S-4 – S-9

Supplementary material and methods

Chemistry characterization

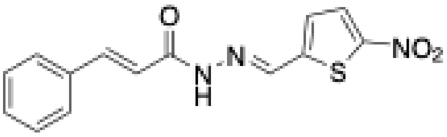

N'-((*E*)-(5-nitrothiophen-2-yl)methylene)cinnamohydrazide

(HI042) Yield: 43%. Solid Orange. mp 246 °C, Purity 99% (HPLC). RMN ^{1}H (300 MHz, DMSO-d6) δ (ppm) 12,1 (s, C=ONH-*), 12,0 (s, C=ONH-*), 8,62(s, N=CH*), 8,32 (s, N=CH*), 8,20 (s, 1H, thiophen-CH), 7,80-7,72 (m, 3H, 2H phenyl-CH; 1H Cβ-H), 7,64 (d, 1H, J= 3,46 Hz, furan-CH), 7,54 (s, 3½H, 3H phenyl-CH, ½H Cα-H*), 6,79 (d, J= 15,6 Hz, ½H, Cα-H*).* corresponding to cis and trans diastereoisomers.

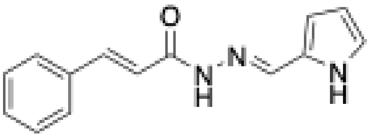

N'-((*E*)-(1*H*-pyrrol-2-yl)methylene)cinnamohydrazide

(HI044) Yield: 34%. Solid Green. mp 231 °C. Purity 99% (HPLC). RMN ^{1}H (300 MHz, DMSO-d6) δ (ppm) 11,5 (s, pyrrole-NH*), 11,4 (s, pyrrole-NH*), 11,4 (s, C=ONH-*), 11,2 (s, C=ONH-*), 8,11 (s, N=CH-*), 7,90 (s, N=CH-*), 7,80-7,57 m, 3½H, 1H Cβ-H, 2H phenyl -CH), 7,47-7,43 (m, 3H, phenyl -CH), 6,99 (s, pyrrole-NCH*), 6,92 (s, pyrrole-NCH*), 6,69 (d, J= 15,8 Hz, ½H Cα-H), 6,50 (s, 1H, pyrrole-CH*), 6,44 (s, 1H, pyrrole-CH*), 6,16 (s, 1H, pyrrole-CH). *corresponding to cis and trans diastereoisomers.

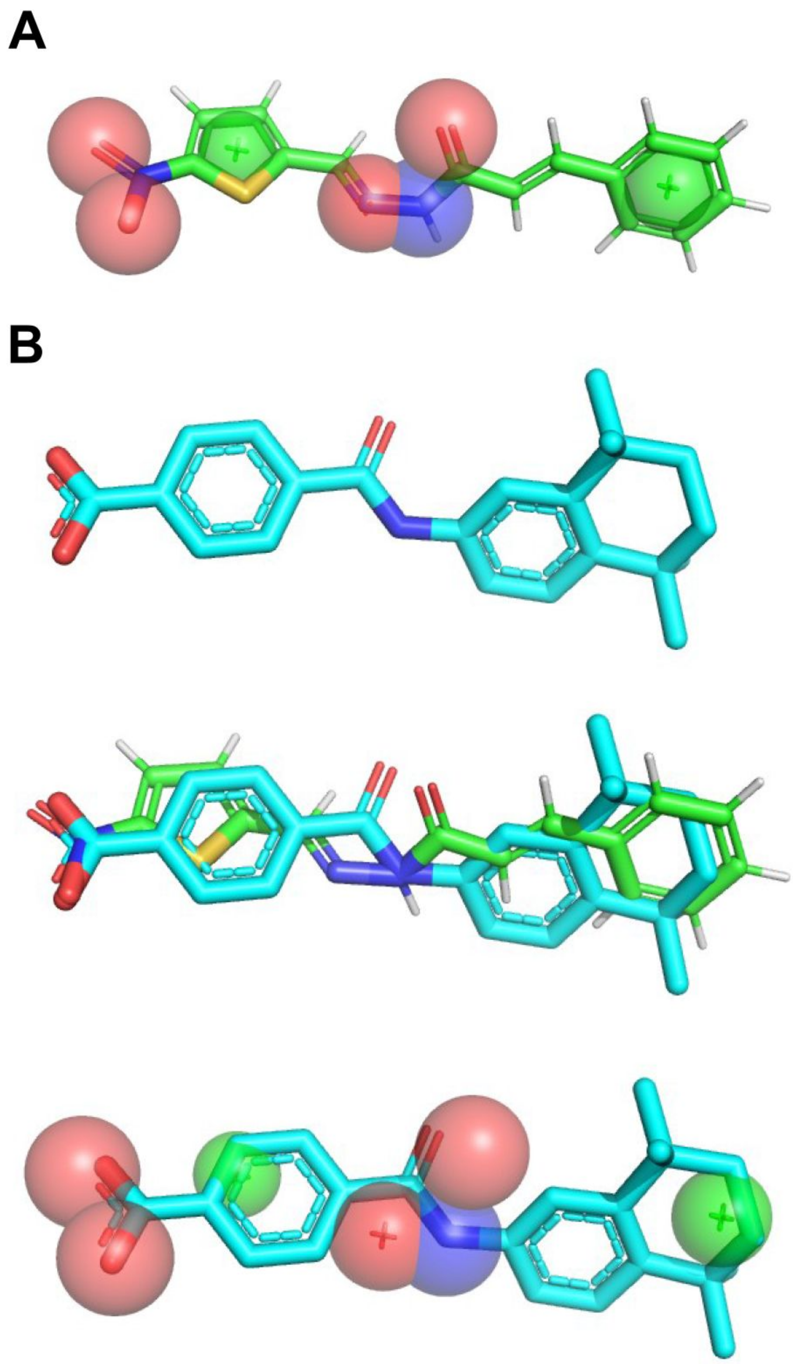

Figure S1. Three-dimensional representation of the HI042 molecule and its pharmacophore, along with its alignment with tamibarotene. (A) Hydrogen bond acceptors regions in red: two oxygen atoms from the nitro group, a hydrazone nitrogen near the double bond, and a carbonyl oxygen. Hydrogen bond donor point in blue: the hydrazone nitrogen/hydrogen near the carbonyl group. Aromatic ring points in green: one for the thiophene ring and another for the benzene ring. **(B)** Tamibarotene aligned with the pharmacophore and HI042. Above, in cyan, is the 3D tamibarotene molecule. In the middle, the tamibarotene molecule is superimposed on HI042 (in green). Below, the molecule aligned with the HI042-based pharmacophore.

Table S1. List of compounds tested and the effects observed in Jurkat and MOLM-13 leukemia cells.

Compound	Structure	MW (g/mol)	Cell viability (% of vehicle-treated cells)			
			Jurkat cells		MOLM-13 cells	
			1 µM	10 µM	1 µM	10 µM
**GA02**	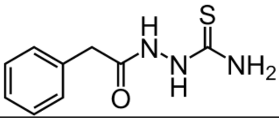	209.27	83.2	76.5	76.8	67.2
**GA04**	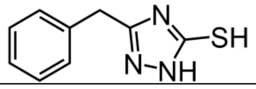	191.25	81.6	71.4	80.7	73.3
**GA10**	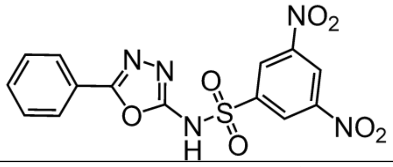	391.32	90.9	85.4	95.2	85.9
**EJC07**	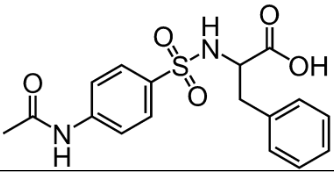	362.40	97.9	81.2	90	71.1
**EJC09**	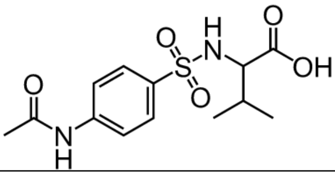	314.36	84.8	75.5	82	68.3
**EJC12**	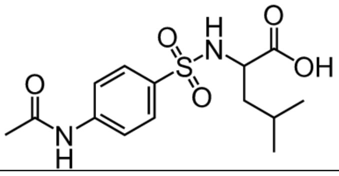	328.38	99.8	87	99.9	82.2
**EJC20**	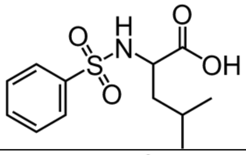	271.33	88.5	82.4	87.5	75.8
**EJC22**	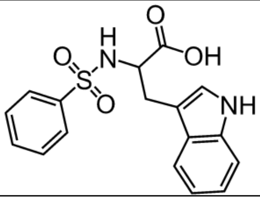	344.38	102.6	85.3	104.8	76.4
**ECAR60**	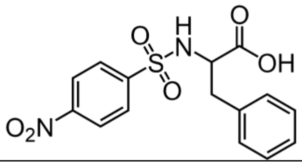	350.35	92.5	81.7	87.2	72.8
**DV07**	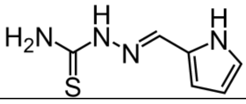	168.22	97.2	82.4	88.2	71.9
**DV10**	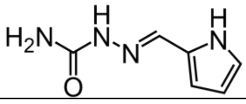	152.15	91.8	85.2	83	75

**DV12**	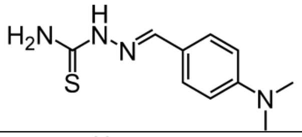	222.31	101.3	79.1	85.3	67.1
**DV13**	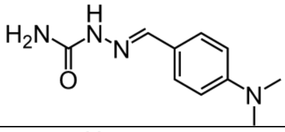	206.24	93.9	81	88.6	73.7
**DV14b**	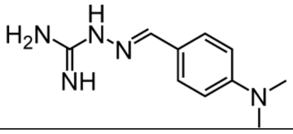	205.26	98	11.9	93.6	36.3
**DV15**	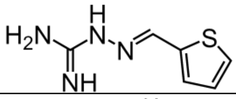	168.22	93.9	89.3	82.5	74.4
**FG009**	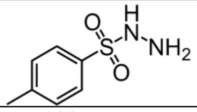	186.23	84.6	73.6	75	63.6
**FG011**	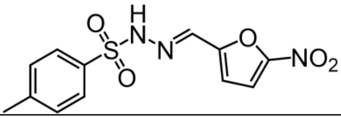	309.30	96.6	84.8	95.1	79
**FG012**	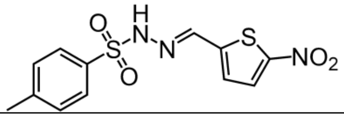	325.36	103.4	104.7	104.9	93
**FG020**	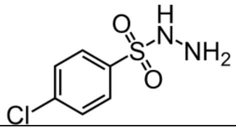	206.65	86	75.4	83.9	66.9
**FG022**	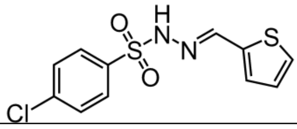	300.78	86.3	76.2	79.8	65
**FG023**	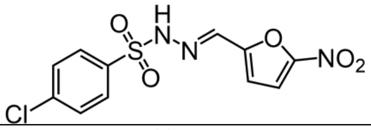	329.72	91.9	79.1	97.9	75
**FG024**	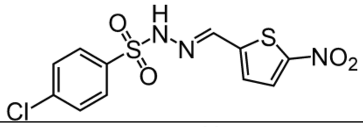	345.78	95.8	99.5	107.6	97.7
**FG026**	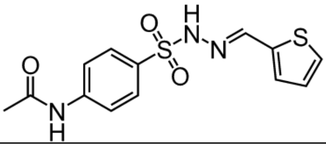	323.39	84.4	77.1	78.1	59.5
**FG027**	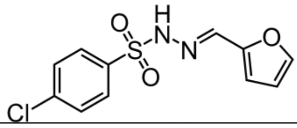	284.72	94.5	85.9	83.3	79.1
**FG028**	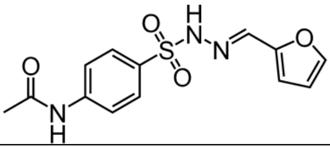	307.33	89	81.2	74.9	63.9

**FG030**	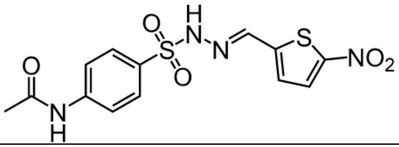	368.39	94.7	92.3	89.3	73.1
**FG031**	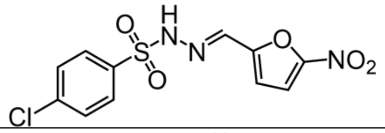	329.72	102.4	93.2	96.6	82
**FG034**	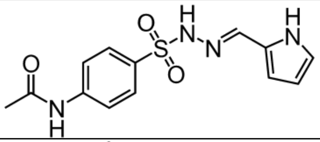	306.34	82.7	75.8	81.3	63.9
**FG038**	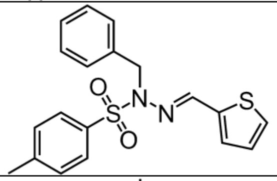	370.49	97.6	88.5	93	84.3
**FG039**	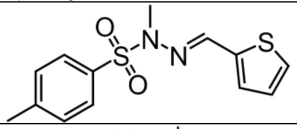	294.39	93.9	84.3	86.1	74.2
**FG042**	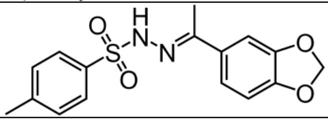	332.37	85	74.4	62.2	59.1
**FG046**	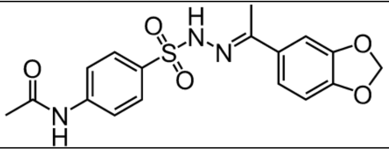	375.40	107	106	103.4	108.3
**FG047**	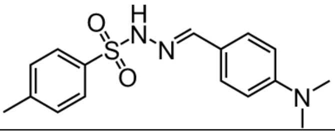	317.41	86.9	81	90.3	71.6
**FG048**	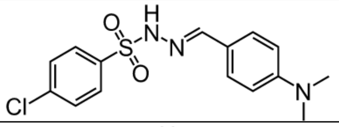	337.82	80.1	72.5	63.4	56.9
**FG049**	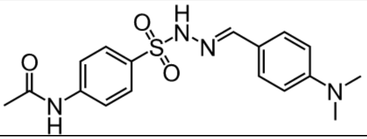	360.43	83	73	63.7	57.6
**FG051**	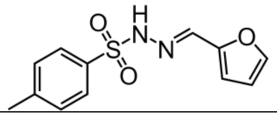	264.30	93.7	86.6	94.9	74
**FG054**	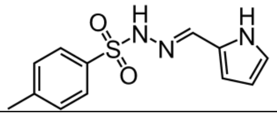	263.32	81.3	72.1	86.8	71.2
**FG055**	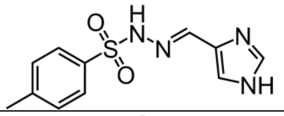	264.30	74.2	66	79.4	62
**FG059**	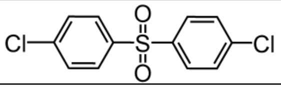	287.16	88	76.4	94	82

**FG061**	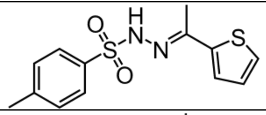	294.39	76.3	72.6	78.9	69.3
**FG062**	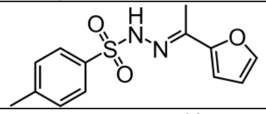	278.33	88.5	82.9	92	73.4
**FG067**	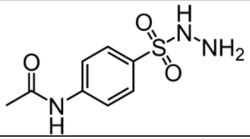	229.26	76.1	69	68.4	58.6
**FG068**	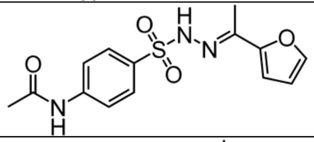	321.35	86.9	76.6	86.7	72.7
**FG072**	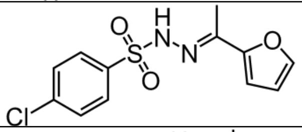	298.75	85.1	76.8	79.4	65.4
**FG073**	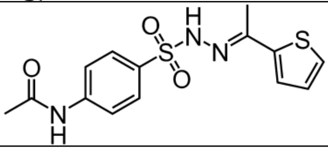	337.42	85.2	74.6	86.6	62.7
**FG075**	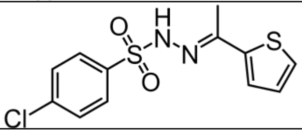	314.81	95	99.2	90.5	80.8
**FG077**	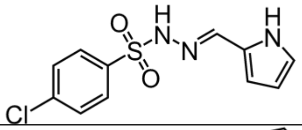	283.73	111	123.3	106.1	105.4
**FG079**	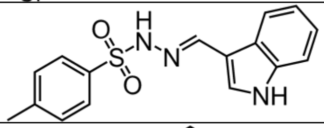	313.37	101.8	80.3	90.1	27.3
**HI023**	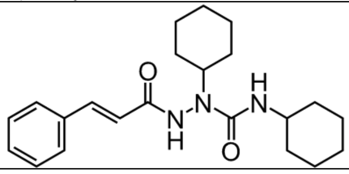	369.50	102.6	98	83.2	78.4
**HI036**	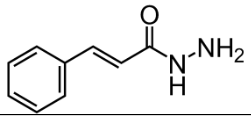	162.19	92.4	84.4	58.1	56.1
**HI038**	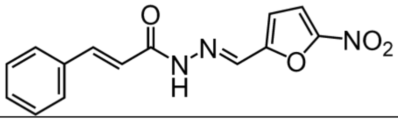	285.25	104.3	90	82.4	59.1
**HI040**	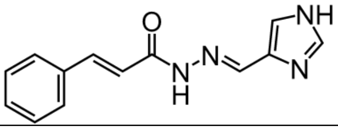	240.26	102.6	9.1	90.1	27.3
**HI041**	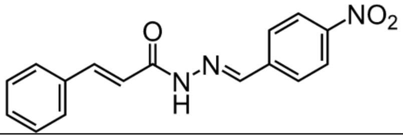	295.29	87.3	84.4	61.5	55.6

**HI042**	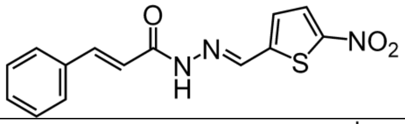	301.32	78.3	52.6	13.6	5.7
**HI043**	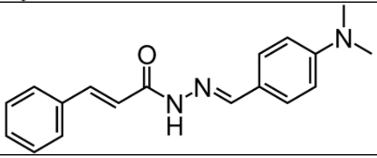	293.36	90.2	61.6	95.1	22.3
**HI044**	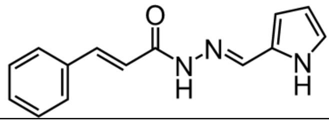	239.27	55	0.7	44	4.4
**HI046**	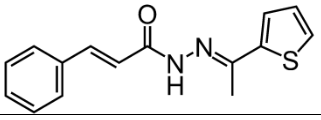	270.35	90	86.9	64	54.7
**HI048**	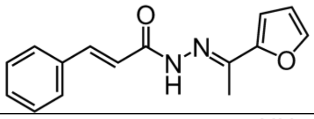	254.28	109.8	91	92.9	79
**HI050**	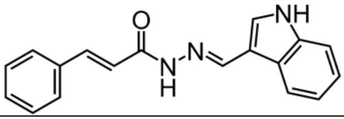	289.33	99	5	98.3	28.4
**HI051**	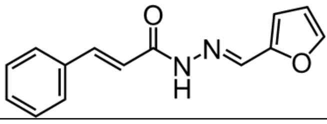	240.26	110.2	92.2	99.7	70.8
**PFM006**	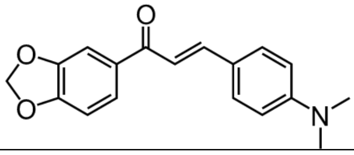	295.33	95.4	1.5	104.4	16.5
**PFM008**	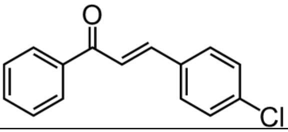	242.70	91.6	17.8	104.9	10.8
**PFM009**	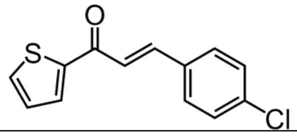	248.73	76.3	48.2	88.7	20.4
**PFM017**	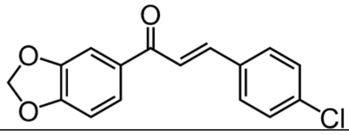	286.71	91.2	73.9	100.4	73.2
**PFM018**	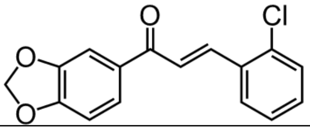	286.71	87.3	68	94.8	80.4
**PFM019**	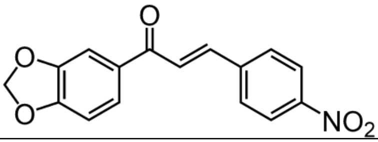	297.26	92.5	75	86.7	70.6
**FM001**	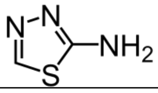	101.13	79.5	72	83	73.7

**FM004**	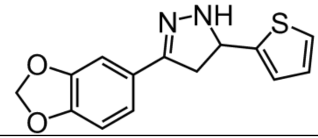	272.32	95.2	87.9	100.2	101.6
**FM010**	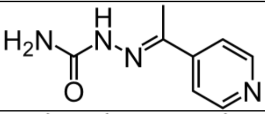	178.19	88	78.8	99.7	80.3
**FM014**	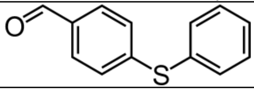	214.28	85.8	80.8	95.9	86.4
**FM016**	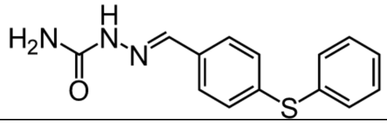	271.34	89.5	76	95.8	82.2
**FM017**	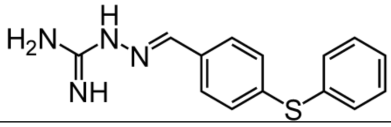	270.35	29.5	0	73.3	0.5
**FM019**	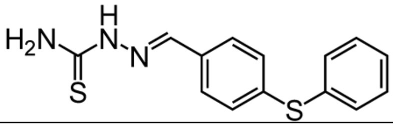	287.40	67.7	1.1	80.1	0.9
**FM026**	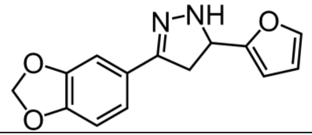	256.26	128.7	120.4	124.6	117.8
**FM027**	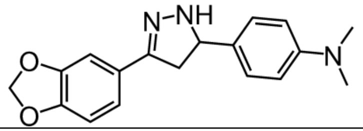	309.36	129	122.3	130.3	100.7
**FM029**	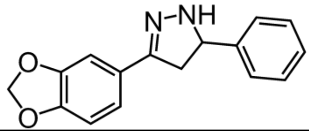	266.29	134.5	87.8	130.1	101.9
**FM031**	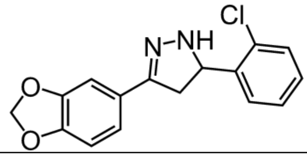	300.74	133.3	110.8	112.7	114.6
**FM033**	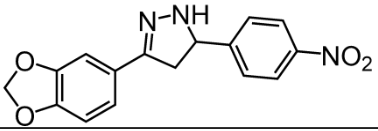	311.29	130.4	94	104.6	81.6
